# Methods for the Economic Evaluation of Health Care Interventions for Priority Setting in the Health System: An Update From WHO CHOICE

**DOI:** 10.34172/ijhpm.2020.244

**Published:** 2021-01-20

**Authors:** Melanie Y. Bertram, Jeremy A. Lauer, Karin Stenberg, Tessa Tan Torres Edejer

**Affiliations:** Department of Health Systems Governance and Financing, World Health Organization, Geneva, Switzerland.

**Keywords:** Cost-Effectiveness Analysis, Economic Evaluation, Health Benefit Package

## Abstract

The World Health Organization’s (WHO’s) Choosing Interventions that are Cost-Effective (CHOICE) programme has been a global leader in the field of economic evaluation, specifically cost-effectiveness analysis for almost 20 years. WHO-CHOICE takes a "generalized" approach to cost-effectiveness analysis that can be seen as a quantitative assessment of current and future efficiency within a health system. This supports priority setting processes, ensuring that health stewards know how to spend resources in order to achieve the highest health gain as one consideration in strategic planning. This approach is unique in the global health landscape. This paper provides an overview of the methodological approach, updates to analytic framework over the past 10 years, and the added value of the WHO-CHOICE approach in supporting decision makers as they aim to use limited health resources to achieve the Sustainable Development Goals (SDGs) by 2030.

## What Is WHO-CHOICE?

The World Health Organization’s (WHO’s) Choosing Interventions that are Cost-Effective (CHOICE) programme has been prominent in the field of economic evaluation, specifically cost-effectiveness analysis for almost 20 years.^1^ According to individual journal metrics, the body of literature produced by WHO-CHOICE has garnered over 3500 citations in the academic literature, 350000 downloads of full text articles and more importantly has contributed to policy discussions in numerous countries as diverse as Argentina, Ethiopia and Estonia^[1]^. Cost-effectiveness analysis plays two roles in the global health landscape. Firstly, it can be used as a quantitative assessment of current and future efficiency within a health system. This supports priority setting processes, ensuring that health stewards know how to spend resources in order to achieve the highest health gain as one consideration in strategic planning.^2^ Secondly, it can be used to support decision-making for new interventions aiming to enter a health benefit package.^3^ Combined; these applications can ensure an optimal use of financial resources within the healthcare sector, ensuring the greatest health gain possible is achieved given the available budgetary space for health.

WHO-CHOICE uses a form of “generalized” cost-effectiveness analysis (GCEA) which aims to support priority setting and efficiency analysis by calculating the most economically efficient health benefit package. This enables the assessment of the allocative efficiency of the current package of healthcare interventions supported in a given setting, and to establish the most efficient potential use of resources into the future. It undertakes this analysis by assuming that all health system constraints can be eliminated, or “bought out” in the long run, and this should be a long term aim of the health system. GCEA is a form of CEA where a hypothetical reference case (“the null”) is used to identify the best package of interventions, regardless of previous, potentially inefficient, decisions. For priority setting, we also relax all health system constraints, the implication being that priority setting assumes there is sufficient capacity within the health system to support any proposed intervention. This represents a scenario where a fully functioning health system with the capacity to support any evaluated intervention exists, and the best value for money package of goods can be developed within that system. This means that no intervention is penalised just because it is being run within an inefficient system, or because past investment decisions were not optimal. This method can show the efficiency with which current and possible new resources are used. WHO-CHOICE evaluates interventions across a range of diseases and risk factors, using a common methodology to allow for comparison and integration of results from single diseases into a sector-wide analysis.^1^

 This paper aims to provide an overview of the methodological approach of WHO-CHOICE and specifically highlight updates made to analytic framework over the past 10 years. As economic evaluation in healthcare is still a young science, and the literature continues to grow, the WHO-CHOICE programme continues to take lessons from the literature and leading experts in the field to ensure that there is added value in the use of the WHO-CHOICE approach in supporting decision-makers as they aim to use limited health resources to achieve the Sustainable Development Goals (SDGs) by 2030.

 The current benefit package in any given setting is often a result of an ad-hoc decision-making process and may be inefficient. GCEA attempts to measure this inefficiency, and guide countries toward the best use of resources. As we enter the SDG era, countries are beginning to transition away from the large global health funding partnerships which were set up to achieve the global goals. Simultaneously, a new range of health conditions targeted under the broad umbrella of achieving universal health coverage (UHC) have received greater prominence in the global landscape. More than ever efficient use of resources is at the heart of the needs of countries in developing their national strategies and designing health benefit packages as they progress toward UHC.

GCEA is a departure from incremental CEA, which can be used as part of a decision-making process when adding to the margins of a package of services already in place in a country. Incremental CEA has become the cornerstone of many country-level decision-making processes through health technology assessment (HTA) agencies.^3^ Incremental CEA makes an implicit assumption when the comparator used is current practice that this represents an efficient use of resources, and usually only two alternative policy options are considered however it is possible for multiple comparisons to be included within a single publication. GCEA, by using a common comparator, allows for many alternatives to be considered simultaneously. Incremental analysis may need to consider health system constraints, such as human resources, infrastructure and logistics which may make the addition of a new intervention not feasible. Within WHO, consideration of such non-budgetary constraints is important but considered within the strategic planning and decision-making process.

The distinction between generalised and incremental cost-effectiveness analysis can be illustrated by means of a simple figure (Figure). The figure represents the usual cost-effectiveness plane, with costs on the x-axis and effects on the y-axis. Compared to the origin, position B is more cost-effective than position A, however in using a different comparator for each intervention, one may consider that both intervention A and intervention B are equally cost-effective. This could represent opportunities for disinvestment in an incremental cost-effectiveness ratio (as a dominant intervention).

**Figure:**
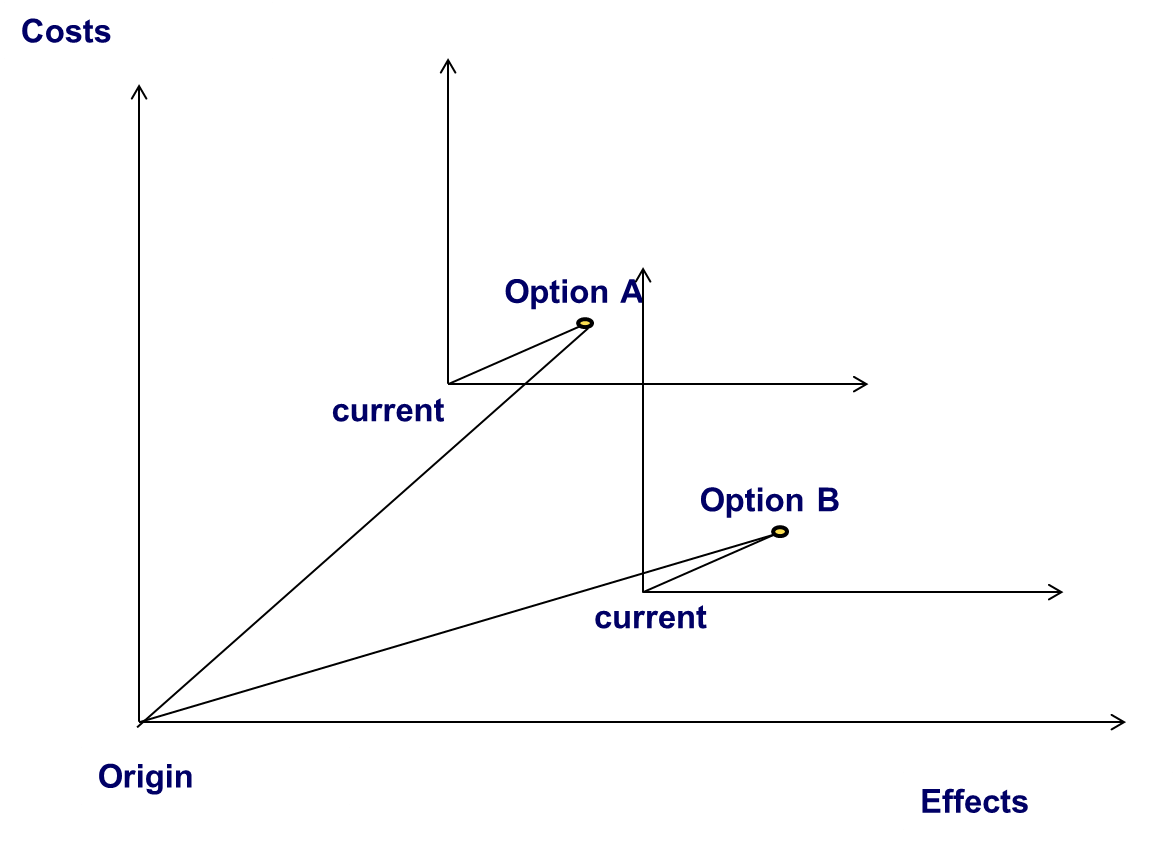
Cost-Effectiveness Plane Indicating the Calculation of Both Average and Incremental Cost-Effectiveness Ratios for Two Interventions.

The cost-effectiveness of position A is different as measured from the current position and as measured from the origin. Measuring from the origin, it is possible to estimate the cost-effectiveness of the current position, giving a measure of the efficiency of the current package. As usual, the origin is defined as the point with coordinates (0, 0). That means here that the origin is the point of no costs and no effects. For this reason, in generalized cost-effectiveness analysis the origin is called the * null position*.

## Methods for WHO-CHOICE Analyses

###  Measurement of Health Impacts

####  Assessing the Effect of Interventions

As in previous WHO-CHOICE analyses, an “intervention” is defined as any preventive, promotive, curative, or rehabilitative action with the primary intention of improving health.^4^ Intervention selection and definition is undertaken in consultation with technical experts from within WHO. In general, interventions can impact health through an effect on any disease rate – incidence, remission or fatality – or by impacting disease severity. Interventions are evaluated first individually compared to the null, and then in combinations to identify the “expansion path,” or the optimal mix of interventions, for each disease area. This is calculated by first starting with the most cost-effective intervention, then sequentially adding interventions to determine the most cost-effective package at every step. The joint effect of interventions, when interventions affect the same rate, is estimated using a multiplicative function. Details of the included interventions are included in each of the disease specific articles within this series. The list of interventions evaluated is not exhaustive and exclusion should not be considered as an implication that an intervention is cost-ineffective.

####  Measuring Population Health Effects

Advancing on previous work using the PopMod population model,^5^ the population health impact models have been largely migrated into the Spectrum platform. This provides consistency between the OneHealth Tool for costing and strategic planning^[2]^ and the WHO-CHOICE models for priority setting. These models project populations at the country level, with proportions of the population moving between health states in accordance with incidence, remission and fatality rates. The time spent in each health state is allocated a health-state valuation using the Global Burden of Disease (GBD) disability weights to measure the health loss.^6^ United Nations Population Division World Population Prospects 2015 were used as the baseline demographic data.^7^ Disease specific epidemiology varies by cause, using a global data source of the nearest year to 2015 available at the time of analysis. Full details are explained in disease specific publications.

## Comparator

The null scenario represents a state whereby no interventions are being delivered for the disease of interest. To calculate the null requires three pieces of data: the epidemiological rate being impacted by the intervention (incidence, remission, case-fatality or disability weight); the effect size of the intervention; the current coverage of the intervention. A simplified example of the calculation used to remove these impacts is shown below (equation 1).^4^


$$\lambda_{N}=\frac{\lambda_{c}}{\left(1-c.e\right)}$$

Where

*λ*_N_ = null hazard rate

*λ*_C_ = current hazard rate

 c = current coverage of intervention

 e = effectiveness of intervention

Where interventions address the same outcome, the multiplicative form of the equation is used (equation 2). Although all disease areas use the same conceptual basis for calculation of the null, full details of the null calibration process are provided in the detailed disease-specific articles in this series.


$$\lambda_{N}=\frac{\lambda_{c}}{\left(1-c_{1}.e_{1}\right)*\left(1-c_{2}.e_{2}\right)*...\left(1-c_{n}.e_{n}\right)}$$

## Measurement of Costs

The overall costing approach follows previously published guidelines on intervention and programme costing for economic evaluation of healthcare interventions.^8,9^ Costs are measured from the health system perspective, regardless of the payer. All costs are measured in 2010 international dollars, and then adjusted to match the baseline epidemiology year using deflators from WHO’s Global Health Expenditure Database. We take an ingredients-based approach identifying all resources required to deliver a healthcare intervention, quantifying the resource requirements (q) and assigning a price to each resource (p). The multiplication of p and q then gives us the cost. Costs are evaluated assuming a constant capacity of the health system, ensuring that differences in cost-effectiveness ratios do not result from interventions being poorly implemented or health system dysfunctions, but rather due to true differences in the costs and effects of interventions.^4^

Costs are divided into patient and programme levels. Patient level costs are those incurred at the point of delivery, and include medicines, diagnostics and health facility visits (including health workforce time). Programme level costs include those costs required to run a health programme, such as administration, monitoring and evaluation, supervision, legislation, training and law enforcement. A common price database and quantity assumptions are used across all disease areas. Full information on data sources and quantity assumptions included in the programme costs have been published elsewhere.^10^ The WHO-CHOICE methodology includes only direct costs and does not include “cost-offsets” ie, the potential future avoided treatment costs associated with an intervention. It also does not include rest-of life treatment costs for those whose lives are saved by intervention implementation.

## Time Horizon

 All interventions and combinations of interventions are evaluated assuming they begin in the year from which epidemiology is drawn, as described above, and continue for the complete required treatment horizon, or life-time, of those impacted. Using a life-time horizon ensures we capture the full population cycle and can compare intervention outcomes across diseases and across prevention and treatment interventions. This represents an update to the methodology used in previous WHO-CHOICE analyses where interventions were evaluated for a 10-year implementation period with the lifelong health impacts projected. The justification for a 10-year implementation period was to fit more closely with planning cycles, and to avoid long range assumptions about epidemiological and demographic changes, continuing health impact and changes in prices. However, it is common practice within the field of economic evaluation to fully capture all costs and benefits associated with an intervention and thus use a life-time horizon. The CHOICE programme now aligns with this common methodological principle.

## Discounting

The results are presented for two scenarios, one which applies a zero-discount rate to health benefits and 3% discount rate to costs, and an alternative scenario using 3% discount rate for both health benefits and costs. This change in results presentation reflects an increasing body of literature around discounting in health economic evaluation, and specifically of the application of a discount rate to health benefits which are not represented in monetary units.^11,12^ A number of economic theories have been used to support the concept of consistency in discounting, with yet further economic, philosophical and ethical theories used to support the concept of differential discounting for monetary and health units.^13^ Given the lack of consensus in the literature the decision was taken for WHO-CHOICE to present scenarios which allow the reader to understand the implication of discounting on establishing priorities, whilst refraining from presenting a methodological choice which may be interpreted as supporting one side of what is seen by the series authors as a healthy scientific debate.

## Reporting of Results

Results are presented as a “cost per healthy life year gained.” Although this is the same measure used in the previous WHO-CHOICE work, disability-adjusted life years (DALYs) as measured by GBD studies^14^ are properly speaking a loss measure and healthy life years measured in cost-effectiveness analysis are a gain measure. To sharpen the distinction between the DALY measure used in GBD analysis, and that used in economic evaluation,^1^ we use the terminology “Healthy Life Year” gained in reporting our results. This analysis maintains the use of the cost per DALY metric for WHO-CHOICE analysis, despite the dominance of cost per QALY as the dominant outcome measure in cost-utility studies in the literature.^15^ This is done for two main reasons. Firstly, cost per DALY is the preferred outcome metric in low- and middle-income settings which is the target audience for this analysis.^15^ Secondly, there is no single database of QALY weights for every disease state and country, which limits the use of this outcome in our analysis. The GBD disability weight survey enables us to use a common data source for valuing health states.^6^

 Due to the enormous amount of inputs and outputs, we do not run probabilistic uncertainty analysis as it is not feasible within our computing system. We instead undertake one-way sensitivity analysis on a number of input values to assess the magnitude of change that is required to lead to a different policy recommendation, given this is the major aim of the WHO-CHOICE programme.

## Thresholds

The use of GDP based cost-effectiveness thresholds to indicate groupings of interventions in previous WHO-CHOICE analyses has generated criticism and debate in the literature.^16^ The GDP based thresholds drawn from the Commission on Macroeconomics and Health were intended as generic global norms used to categorise interventions into broad groups for consideration within a local context.^17,18^ Unfortunately these were seen by some as generic decision rules to be used in HTA-type processes.^16^ A recent publication clarifies the role of cost-effectiveness results in the decision-making process from the WHO-CHOICE perspective and consequently recommends against using cost-effectiveness threshold as a decision rule, but rather using cost-effectiveness analysis as part of a transparent and fair decision-making process.^2,18^ For ease of interpretation, the current results are presented only in league tables or log-log scale graphs showing results by multiples of 10. No threshold is used to define interventions as cost-effective or not. By using GCEA, and a common comparator with the null, we make clear in our results the trade-offs in deciding between different interventions, by highlighting in a quantifiable manner the opportunity cost of alternative investment decisions.

## Using Cost-Effectiveness Information to Support Priority Setting and Decision-Making

Using cost-effectiveness information in priority setting and decision-making is challenging, but necessary in order to increase efficiency of health system spending whilst working toward UHC.^19^ Cost-effectiveness ratios are undoubtedly informative in assessing value for money. However, they need to be considered alongside other quantitative measures such as feasibility and budget impact, as well as value-based considerations such as fairness in decision-making processes. Countries should consider establishing a *context-specific process* for decision-making that is supported by legislation, have stakeholder buy-in and are transparent, consistent and fair. WHO-CHOICE strives to provide global level evidence to support priority setting, with the option of tailoring results to country level using a contextualisation platform. To support this, we have developed a new country contextualisation tool, CHOICE-Spectrum, which enables faster, more user friendly in-country processes than previous WHO-CHOICE tools. Methods used in the CHOICE-Spectrum tool adhere to the methods presented in this article. The tools are freely available for download (https://www.who.int/choice/en) and are supported through user manuals, with technical assistance and peer review options available to WHO Member States wishing to utilise the tools.

 CHOICE-Spectrum provides countries with the opportunity to quickly develop locally contextual evidence to begin an evidence supported priority setting activity, to develop a health benefits package or to create a database of cost-effectiveness results for use in an HTA decision-making process. Further, an interface between CHOICE cost-effectiveness results and the United Nations supported-OneHealth Tool for strategic planning allows a streamlined approach to economic evaluation and scenario analysis for estimating the financial and non-financial requirements to implement a health strategy. This aligns with growing demand from WHO Member States for support to develop HTA processes and strengthen strategic planning processes for UHC, with an emphasis on country-owned objective, quantitative measures to support both practises.

 In the last 15 years, cost-effectiveness analysis has seen major advances in theory and empirical knowledge, and WHO-CHOICE has benefited from many of these contributions. Concurrently, effective intervention options have expanded, and prices have continued to change. An update and expansion of WHO-CHOICE is timely, and the release of accessible tools for rapid national contextualisation processes can support countries as they look to meet the challenges of the SDG era.

## Ethical issues

 No ethical approval was sought as this is a secondary data analysis.

## Competing interests

 Authors declare that they have no competing interests.

## Authors’ contributions

 MB coordinated the update of the WHO-CHOICE analyses including technical inputs on the methods changes and development of the linked toolkit, and drafted this manuscript. JAL provided technical input into all methods updates and edited the manuscript. KS provided technical input into costing methodologies and development of the linked toolkit and edited this manuscript. TTTE oversaw the development of new methodology, contributed to the framing of the work and has edited the manuscript.

## Disclaimer

 MYB, JAL, KS and TTTE are staff members of the WHO. The views expressed in this paper are solely the responsibility of the named authors and do not necessarily reflect the decisions or stated policy of the WHO or its Member States.

## Funding

 This study was funded by the WHO.

## Endnotes

 [1] Numbers taken from journal metrics for each individual methods and results article previous published on WHO-CHOICE and combined by the authors.
[2] http://www.avenirhealth.org/software-onehealth.php.

